# Contamination Status and Ecological Security Thresholds of Fluoride in Farmland around a Phosphorus Chemical Plant in a Karst Area of Southwestern China

**DOI:** 10.3390/toxics11070587

**Published:** 2023-07-05

**Authors:** Ziyu Guo, Min Wang, Hengmei Dai, Sha Pan

**Affiliations:** The Key Laboratory of Environmental Pollution Monitoring and Disease Control, School of Public Health, Ministry of Education, Guizhou Medical University, Guiyang 550025, China; ziyuguo@foxmail.com (Z.G.); minwang@foxmail.com (M.W.); 18798057609@163.com (H.D.)

**Keywords:** fluoride, health risk, ecological security threshold, phosphorus chemical plant, karst area

## Abstract

The phosphorus chemical plant (PCP) production process leads to the substantial discharge of fluoride into the surrounding environment. However, there is limited research data regarding the pollution levels and ecological safety thresholds of farmland fluoride around PCPs in karst areas. This study evaluates the local contamination status and health risks by determining the fluoride content in farmland and vegetables in vicinity of a PCP in a karst area of Southwestern China. Farmland ecological security threshold was derived based on the species sensitivity distribution (SSD) model. Results showed that the fluoride contents in the soil ranged from 529.36 to 1496.02 mg kg^−1^, with the median value of 823.93 mg kg^−1^, which was higher than the national background value in China (478 mg kg^−1^) by 172.37%. Fluoride contents in vegetables ranged from 0.01 to 25.21 mg kg^−1^, with the median value of 1.29 mg kg^−1^, which was higher than the limits of contaminants in food (1 mg kg^−1^) by 129.00%, and 53.85% of vegetable samples were contaminated. Leafy vegetables showed a higher tendency of fluoride enrichment compared to non-leafy vegetables. Despite there being no significant health risk to the residents via the intake of vegetables overall, there may be potential health risks from the intake of sweet potato leaves. Based on the SSD model, the ecological safety thresholds of fluoride in local farmland was classified into the suitable for planting category (≤174.13 mg kg^−1^), safe plant utilization category (174.13–4005.42 mg kg^−1^), and strict control of planting category (≥4005.42 mg kg^−1^). These findings will provide valuable insights to facilitate the safe cultivation of local agricultural products and optimize the utilization of soil resources.

## 1. Introduction

Karst is a unique geological topography that is formed from soluble rocks, such as limestone, doloite, and gypsum. Soil erosion and ecological fragility in karst areas are severe and extremely vulnerable to human activities [1]. The production raw material, phosphate ore, contains a high concentration of fluorine [2]. Therefore, phosphorus chemical plant (PCP) release significant amounts of fluoride into the surrounding environment during the production process, which has become a major source of environmental fluoride in karst areas [3].

Fluorine is a trace element ubiquitously distributed in soil, rocks, water, air, and biota [4]. Fluorine is a crucial micronutrient for human wellbeing and exhibits both beneficial and detrimental effects on human health. Adequate fluorine consumption can be beneficial to teeth and bone health; however, excessive body intake of fluorine develops various human health problems, such as dental fluorosis, skeletal fluorosis, and renal and hepatic impairment [5].

Generally, drinking water has been identified as the principal cause of fluorosis in humans [6]. In several regions, including Algeria, China, Egypt, India, and Iran, human populations have been observed to experience fluorosis resulting from the consumption of water with high fluoride content [7]. Additionally, the use of coal that is rich in fluoride for cooking and heating can also result in excessive fluoride accumulation in the body [8]. In recent years, food has also been deemed as a non-negligible route for human fluoride intake [9]. The sources of fluoride in food are complex and diverse, including the fluoride content present in the soil where vegetables and crops are cultivated, irrigation with water-containing fluoride, and the direct deposition of fluoride from the atmosphere onto the surfaces of vegetables and crops [10]. In general, weathering and dissolution of fluoride-containing minerals are considered to be major sources of fluoride in environments such as soil and water [11]. However, anthropogenic fluoride release into the environment increased continuously owing to the rapid industrial development of the past few years [12]. Some industrial activities, such as steel, aluminum, glass, and fertilizer manufacturing, are considered important anthropogenic sources of fluoride pollution in the environment [3]. During the production of phosphate fertilizer, certain amounts of fluorides are known to release into the air and subsequently accumulated on the surface soil, and the highest content of fluoride is observed at the distance of up to 2 km from the plant [13]. Krełowska-Kułas [14] reported that the fluoride contents in vegetables grown near a steel plant were 0.39–8.82 mg kg^−1^ and were higher than that of control areas. Vegetable and soil samples in close proximity to the zinc smelter exhibited fluoride contents ranging from 0.36 to 0.71 and 101.67 to 189 mg kg^−1^ [15]. Potential health risks of fluoride exposure from the consumption of vegetables by residents can be due to absorption by plant roots from contaminated soil, and the direct deposition of fluoride onto plant surfaces from the atmosphere [16]. Therefore, soil and vegetables in the vicinity of the industry may pose a serious risk to the health of residents.

To guarantee food safety and regulate the rational utilization of agricultural soil, several countries such as Japan, Canada, and the United States have established soil fluoride threshold guidelines. However, China has not yet specified the limit values for soil fluoride [17]. In numerous countries, the soil quality limit values employed for agricultural purposes rarely considered different soil types and crop cultures with varied accumulation capacities of the element. Some studies have found that the exceedance rate of fluoride in soil is often different from that in the vegetables produced in it, which indicates that the current soil fluoride quality standards cannot fully guarantee vegetables’ quality and safety [17,18]. Therefore, it is crucial to establish precise soil fluoride thresholds to ensure the secure administration of soil in areas dedicated to agricultural production. In recent years, soil ecological security thresholds derived from a combination of food quality standards and soil-vegetable transfer models are considered to be more reasonable, avoiding overprotection or inadequate protection and saving economic and ecological costs [19]. Multiple models have been applied to the derivation of soil ecological security thresholds. Among these models, the species sensitivity distribution (SSD) model, which takes into account bioaccumulation factor (BAF) of the soil-crop paired sites, has gained widespread popularity. The popularity of this model stems from the fact that it considers the variations in species sensitivity, soil physical and chemical characteristics, bioavailability, and sources of pollutants [20,21].

To sum up, the research data on the pollution situation and ecological security threshold of farmland fluoride around the PCP in the karst area is limited. Thus, we hypothesize that phosphorus chemical production may result in fluoride contamination of the surrounding farmland soil and vegetables, posing potential risks to human health. Accordingly, the objectives of the current work are: (1) to determine the levels of fluoride in farmland and vegetables in the vicinity of a PCP in a karst region; (2) to evaluate the potential human health risks via the consumption of local vegetables; (3) to derive the ecological safety thresholds of soil fluoride in the study area based on the SSD model.

## 2. Materials and Methods

### 2.1. Study Area

**Figure 1:**
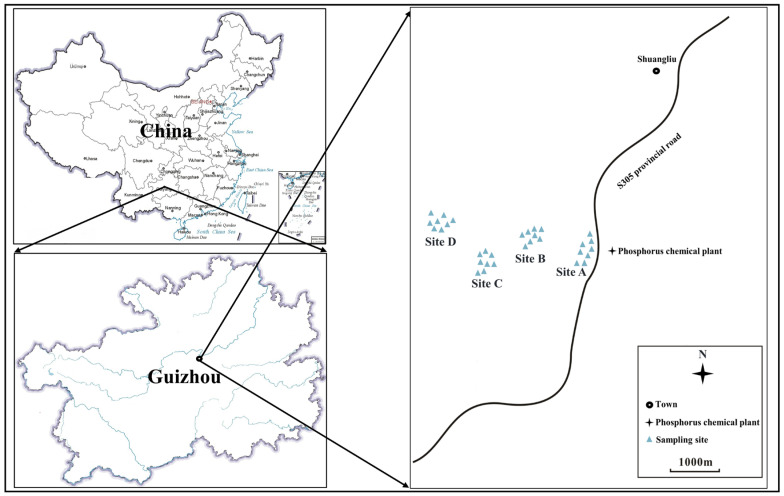
Location of sampling sites.

The study area is situated in Kaiyang county, Guizhou province, Southwestern China, at the geographical coordinates of 27°0′41.83″ N–27°1′15.86″ N and 106°47′59.61″ E–106°48′57.21″ E and has a characteristic karst topography (Figure 1). The climate is influenced by the subtropical monsoon, with a dominant wind direction from the northeast. The mean annual temperature is 13.49 °C, and annual precipitation is 1258 mm, with hot and humid summers and cool and dry winters. Kaiyang County is rich in phosphate resources. It is one of the largest phosphate rock bases in China, has many PCPs, and the local economy is mainly dependent on phosphate. The PCP in this study is one of the largest plants in Kaiyang County.

### 2.2. Sample Collection and Analysis

In this study, four sampling sites were set up at different distances within 3 km of a PCP based on geographic and meteorological conditions, i.e., 0.5 km (sampling site A), 1 km (sampling site B), 2 km (sampling site C), and 3 km (sampling site D) from the PCP in the prevailing downwind direction. Eight sampling locations were set up at each sampling site, and each natural farmland was one sampling location. Soil samples were collected from a total of 32 locations using the plum blossom multi-point mixed sampling method at a depth of 0–20 cm below the soil surface [22].

The vegetable samples were also collected in 32 locations [23]. In total, 91 vegetable samples, representing 48 leafy vegetables, 24 melon and fruit vegetables, and 19 root vegetables, including 16 vegetable species such as pakchoi (*Brassica Chinensis* L.), eggplant *(Solanum melongena* L.), and potato (*Solanum tuberosum* L.), etc., which are commonly grown in the study area, were taken from the plants growing in the sampled soil (Table 1). Among them, separate vegetable sample types were considered for sweet potato leaves, sweet potato roots, pumpkin leaves, and pumpkin fruits, due to their wide consumption within the region [24]. For each sample at each sampling location, at least three replicates were collected to form a composite sample. Soil and vegetable samples were placed into polyethylene bags and then brought to the laboratory for processing and analysis immediately.

The soil samples were subjected to air-drying at ambient temperature. Upon drying, any visible debris and larger rocks were eliminated, and the remaining soil was finely ground until it could pass through a sieve with a mesh size of 0.15 mm. Soil pH was measured in 1:5 soil/water suspension. Total fluoride of soil was determined by following the method of China environmental protection standard HJ 873-2017. Briefly, 0.20 g of soil sample was mixed with 2.00 g of NaOH in a nickel crucible and heated in a muffle furnace. It was heated at 300 °C for 10 min, then raised to 560 ± 10 °C for 30 min. After cooling, the mixture was dissolved in hot water (80–90 °C) and transferred to a 100.00 mL cuvette. Then, 5.00 mL of hydrochloride (1:1) was added, the volume was adjusted to 100.00 mL using deionized water and left to stand before testing. The fluoride content was measured using a fluoride ion-selective electrode (CSB-F2, Changsha Yiming, Changsha, China) [25].

The edible parts of vegetables were first washed in running tap water, then rinsed with deionized water, and finally dried to constant weight in oven at 60 °C. The fresh and dry weights of vegetables were measured. All vegetables were then milled to pass through 0.18 mm mesh sieve and kept for fluoride determination by the Chinese standard method of fluoride analysis in food (GB/T 5009.18-2003). Briefly, 1.00 g vegetable sample was extracted by 10.00 mL of hydrochloride (1:11) for 1 h at room temperature with occasional gentle shaking. The resulting extract and the standard solution (fluoride) were then mixed with 25.00 mL total ionic strength adjusting buffer, and the volume was adjusted to 50.00 mL using deionized water. The fluoride content of the mixture was measured using a fluoride ion-selective electrode (CSB-F2, Changsha Yiming, Changsha, China) [26].

### 2.3. Contamination Assessment of Soils

The total fluoride index method was used to evaluate the soil fluoride environmental quality [27,28], as in the equation below:

(1) $$P_{i}=C_{i}/S_{i}$$

where P_i_ represents the soil fluoride environmental quality index, C_i_ is the measured value of fluoride content in soil (mg kg^−1^), and S_i_ is the evaluation standard of fluoride in soil (mg kg^−1^).

The background value of fluoride content in surface soil in China (478 mg kg^−1^) and the mean value of fluoride content in soil (800 mg kg^−1^) in the area where endemic fluorosis occurs are the standard values (S_i_) for evaluation [28], the grading standards are shown in Table 2.

### 2.4. Bioaccumulation Factor (BAF)

The bioaccumulation factor (BAF) was employed to assess the transfer characteristic of soil-vegetable paired samples [24], as in the equation below:

(2) $$\mathrm{BAF}=C_{\mathrm{vegetable}}/C_{\mathrm{soil}}$$

where C_vegetable_ and C_soil_ are the content of fluoride in vegetables and their corresponding soil, respectively (mg/kg).

### 2.5. Health Risk Assessment of Fluoride through Vegetables

#### 2.5.1. Estimated Daily Intake

Estimated daily intake (EDI) of the fluoride via vegetable consumption was calculated using the following equation:

(3) $$\mathrm{EDI}=\frac{[C]\times W}{\mathrm{body}\;\mathrm{weight}}$$

where the [C] is the content of fluoride in vegetables (mg kg^−1^), the daily ingestion rate of vegetables (W) for adults and children (1–14 years old) were 345 and 232 g person^−1^ day^−1^, respectively [29], and the body weights of adults and children were taken as 63.1 and 33 kg, respectively [30]. 

#### 2.5.2. Health Risk

The health risk of fluoride exposure via vegetable consumption was determined by calculating the ratio of the EDI of fluoride to the reference dose (RfD), as in the equation below:

(4) $$\mathrm{Risk}\;\mathrm{index}=\frac{\mathrm{EDI}}{\mathrm{RfD}}$$

where RfD represents safe levels of exposure orally during a lifetime. The USEPA [31] derived RfD of 0.06 mg kg^−1^ day^−1^ for fluoride. If the risk index is less than 1, it indicates that there is no significant health risk associated with the intake of fluoride through vegetable consumption. In contrast, if the risk index is equal to or greater than 1, it suggests that the exposed population may face health risks, requiring the implementation of appropriate interventions and protective measures.

### 2.6. Derivation of Soil Ecological Security Threshold

The vegetable samples were arranged in descending order according to the bioaccumulation factor of fluoride. The corresponding ordinal number (R) was set according to the order, and the cumulative probability value (P) was calculated [22], as in the equation below:

(5) P=R/(N+1)

where P represents the cumulative probability value corresponding to each vegetable, R is the sequential number assigned to each vegetable in descending order based on their BAF, and N is the number of vegetable species.

The distribution of Log-logistic were applied to fit the curves of species sensitivity distribution (SSD) based on the 1/BCF data and cumulative probability values. The equation for Log-logistic function is as follows:

(6) $$y=\frac{a}{1+({\frac{x}{X_{0}})}^{b}}$$

where y represent the cumulative probability value corresponding to vegetables (%), x is the 1/BAF, and a, b and X_0_ are fitting parameters.

Based on the SSD curve fitted with the Log-logistic distribution model, the 1/BAF values corresponding to 80%, 50%, and 20% of the vegetable species protected were calculated. Furthermore, the ecological security thresholds of fluoride in soil were derived according to the National Food Safety Standards of China (GB 2762-2017) [21], as in the equation below:

(7) $$C_{\mathrm{soil}}=C_{s}/(1/{\mathrm{HC}}_{p})$$

where C_soil_ represents the ecological security thresholds of fluoride in soil (mg kg^−1^), C_s_ is the limit of fluoride in vegetables in the national standard (mg kg^−1^), HC_p_ is the 1/BAF value for protecting 80%, 50%, and 20% of vegetable species [20,32].

### 2.7. Quality Control

In order to mitigate the impact of extraneous variables on the experimental samples, appropriate quality assurance procedures and precautions were employed. The national standard material (GBW07405 (GSS-5) for soil and GBW10020 (GSB-11) for vegetable) was used for quality control of fluoride content analysis during sample determination. The recovery rate of the sample was controlled in the range of 90–110%. Every 10 samples set parallel samples, with relative deviation control within 10%, and blank samples were also included in each batch for analysis.

### 2.8. Statistical Analysis

The statistical software package SPSS 16.0 was employed for data analysis. The Shapiro–Wilk normality test with a 95% confidence level was used to examine the distributional normality of the data. Since the data were not normally distributed, subsequent nonparametric statistical analyses were employed. The Kruskal–Wallis test was utilized to detect significant differences in the fluoride content, pollution index, BAF value, and risk index. Linear regression analysis was performed to establish relationships between fluoride contents in soil and distance from the emission source. The fitting of the SSD curve and the drawing of the graph were conducted using the Origin 2019b.

## 3. Results and Discussion

### 3.1. Fluoride Content in Soil

**Figure 2:**
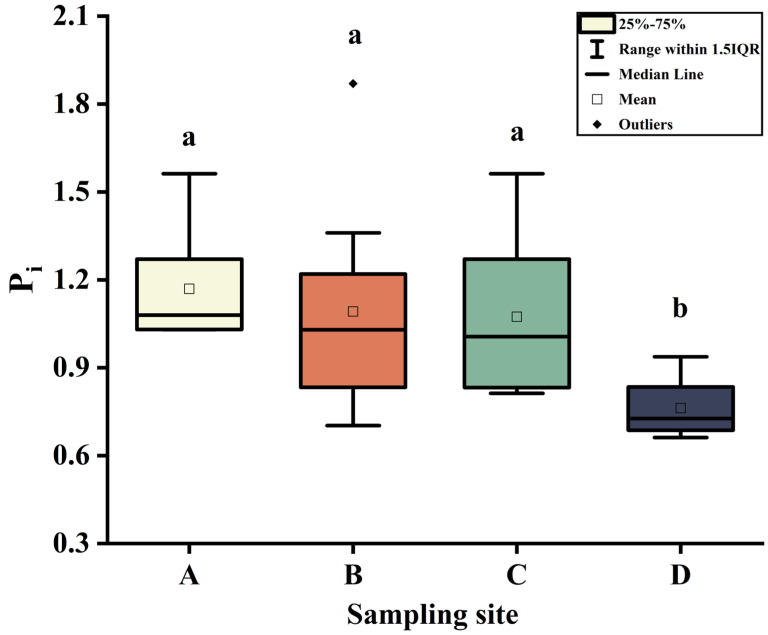
Evaluation of soil environmental quality for fluoride pollution at different sites. Sampling sites A, B, C, and D were situated at distances of 0.5, 1, 2, and 3 km, respectively, downwind from the phosphorus chemical plant (PCP). Boxes identified by the different letters are significantly different (*p* < 0.05).

The pH and content of fluoride in the soil is presented in Table 3. The soil pH ranged from 3.97 to 7.40, i.e., strongly acidic to mildly alkaline. The fluoride contents in soil varied from 529.36 to 1496.02 mg kg^−1^, with the median value of 823.93 mg kg^−1^. This value was higher than both the national background value in China (478 mg kg^−1^) and the fluoride content in endemic fluorosis areas of China (800 mg kg^−1^) by 172.37% and 103.00%, respectively [28]. The evaluation of soil environmental quality for fluoride pollution is presented in Figure 2. The P_i_ values of sample sites A, B, C, and D were 1.080, 1.030, 1.007, and 0.726, respectively. All sample sites reached the pollution level except sample site D. The results suggest that the soil in this study area was generally polluted with fluoride, probably due to the activity of the PCP. Phosphate ore, a crucial raw material for the phosphorus chemical industry, is characterized by its high fluoride content [33]. The anthropogenic activities associated with PCPs have led to significant accumulation of fluoride in the surrounding soil, as evidenced by numerous studies conducted in various regions [12,34].

**Figure 3:**
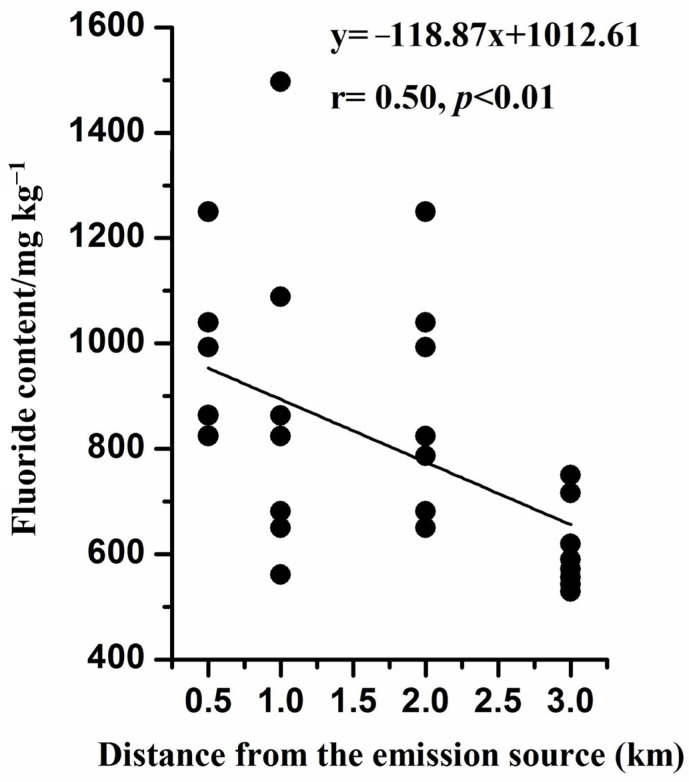
Fluoride content in soil related to the distance from the emission source.

The fluoride in the soil was negatively correlated (r = 0.50, *p* < 0.01) with the distance to the pollution source (Figure 3). Higher contents for the fluoride were observed in locations closer to the PCP, probably ascribed to the distance of sampling points from the pollution source as well as the wind direction, etc. A similar trend has also been reported where the fluoride content at the surface soil is a function of the distance from an aluminum industrial plant [35].

### 3.2. Fluoride Content in Vegetables

**Figure 4:**
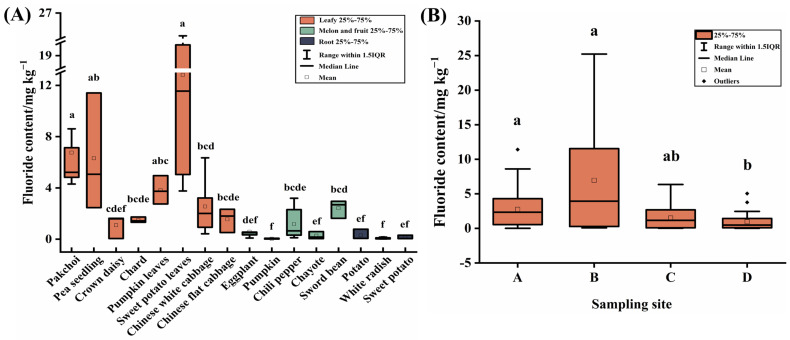
Fluoride contents in vegetables (mg kg^−1^, fresh weight). (**A**) Different species of vegetables, (**B**) Different sample sites, sampling sites A, B, C, and D were 0.5, 1, 2, and 3 km from the PCP in the prevailing downwind direction, respectively. Boxes identified by the different letters are significantly different (*p* < 0.05).

The fluoride contents in the edible part of different vegetables are presented in Figure 4A. The range of fluoride contents in vegetables was 0.01 to 25.21 mg kg^−1^ (median value, 1.29 mg kg^−1^). Based on the limits of contaminants in food (GB 2762-2005), the median value of fluoride content in vegetables was higher than the limit value (1 mg kg^−1^) by 129.00%, and 53.85% of vegetable samples exceeded this limit. This finding is similar to the studies reported by Mezghani et al. [36] where large quantities of fluoride was accumulated by vegetation growing in vicinity of the factory. In the soil-vegetables system, vegetables efficiently absorb free fluoride ions, leading to the accumulation of fluoride, thus making vegetables a significant contributor to human exposure to fluoride [27]. The present results imply that the health risks caused by vegetable ingestion cannot be overlooked. 

For the fluoride contents in different vegetables, leafy vegetables had 0.06–25.21 mg kg^−1^ (median value, 2.90 mg kg^−1^), melon and fruit vegetables had 0.01–3.20 mg kg^−1^ (median value, 0.47 mg kg^−1^), root vegetables had 0.02–0.78 mg kg^−1^ (median value, 0.08 mg kg^−1^), excessive rate were leafy vegetables (85.42%) > melon and fruit vegetables (33.33%) > root vegetables (0.00%). The fluoride contents of leafy vegetables are notably higher than that of non-leafy vegetables (*p* < 0.05). Among them, four leafy vegetables (sweet potato leaves, pakchoi, pea seedling, and pumpkin leaves) were the most seriously contaminated with fluoride, with median values of 11.55, 5.22, 5.07, and 3.75 mg kg^−1^, respectively (*p* < 0.05). He et al. [27] reported that vegetables have different degrees of fluoride enrichment, with leafy vegetables showing higher levels of fluoride enrichment, which is consistent with the result of this study. Leafy vegetables are more vulnerable to pollutant accumulation due to their high foliar surface areas and higher translocation, transpiration efficiency, and fast growth rates [37,38,39].

For different sample sites, the median values of the fluoride content in vegetables for sites A, B, C, and D were 2.34, 3.94, 1.16, and 0.48 mg kg^−1^, respectively (Figure 4B). The fluoride contents in sites A and B were significantly higher than those in site D (*p* < 0.05), but there were no statistically significant differences between sites A and B, which is consistent with the results of fluoride contamination assessment in soils. This result suggests that the contamination caused by the activity of PCP significantly decreases at locations 3 km from the factory.

**Figure 5:**
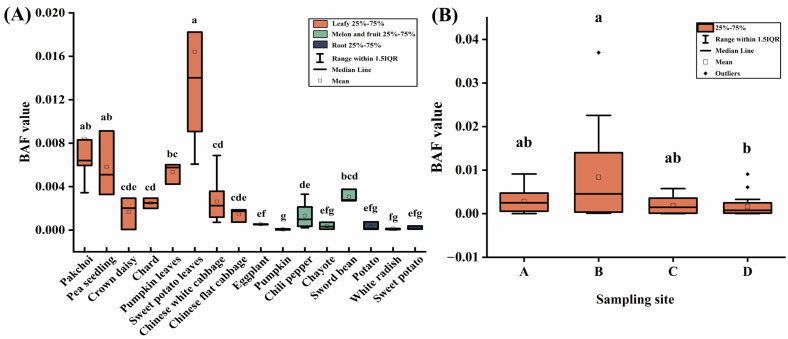
The BAF value of vegetables for fluoride. (**A**) Different species of vegetables, (**B**) Different sample sites, sampling sites A, B, C, and D were 0.5, 1, 2, and 3 km from the PCP in the prevailing downwind direction, respectively. Boxes identified by the different letters are significantly different (*p* < 0.05).

Fluoride migrates from the soil and air to vegetables, where it accumulates. Subsequently proceeds through the food chain to reach the human body, posing a significant risk to human health. The BAF was used to evaluate the migration ability of fluoride from soil to vegetables. The BAF value of vegetables for fluoride in their soil is shown in Figure 5A. The BAF values of vegetables for fluoride ranged from 0.02 × 10^−3^ to 3.70 × 10^−2^ (median value, 0.17 × 10^−2^). For different vegetables, the BAF values were leafy vegetables (median value, 0.33 × 10^−2^) > melon and fruit vegetables (median value, 0.06 × 10^−2^) > root vegetables (median value, 0.01 × 10^−3^) (*p* < 0.05). Among them, four leafy vegetables (sweet potato leaves, pakchoi, pumpkin leaves, and pea seedling) had a stronger enrichment ability to fluoride, with median BAF values of 1.40 × 10^−2^, 0.64 × 10^−2^, 0.57 × 10^−2^, and 0.51 × 10^−2^, respectively (*p* < 0.05). The different BAF values of vegetables in this study indicate that each variety has a unique response to fluoride contamination; this agrees with the results previously reported by Wang et al. [40]. This disparity could primarily be associated with the physiological traits of diverse vegetables. In general, vegetables with high BAF values for fluoride have longer root lengths, more root tips, and a larger leaf surface area [24]. 

For different sample sites, the median BAF value of fluoride for sites A, B, C, and D were 0.25 × 10^−2^, 0.46 × 10^−2^, 0.15 × 10^−2^, and 0.08 × 10^−2^, respectively (Figure 5B). There was no statistically significant difference in the BAF values of fluoride between site A, B, and C. Site B had significantly higher BAF value of fluoride than site D (*p* < 0.05), which is generally consistent with the results for fluoride contents in vegetables.

### 3.3. Health Risk Assessment of Fluoride Intake from Vegetables

**Figure 6:**
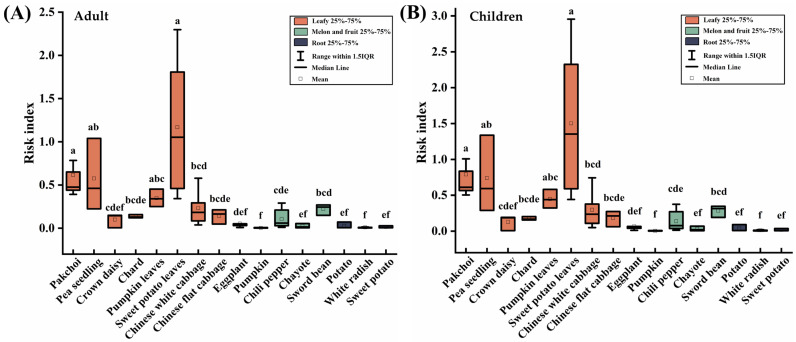
Health risks of vegetables with fluoride intake. (**A**) Adult, (**B**) Children. Boxes identified by the different letters are significantly different (*p* < 0.05).

For a considerable period of time, it has been widely acknowledged that the occurrence of fluorosis is linked to the heightened concentration of fluoride in drinking water. Nevertheless, it is worth noting that vegetables with a substantial fluoride content also make a significant contribution to overall fluoride consumption [10]. The risk index has been recognized as a valuable parameter for assessing the potential risks related to the consumption of food crops contaminated with excessive fluoride levels [7,34]. The risk index of fluoride was calculated for different vegetables and is presented in Figure 6. The overall risk index of fluoride for adults and children were 0.117 and 0.151, respectively, which were all less than 1. This result implies that if residents’ intake of fluoride is only from vegetables, they may not be subject to a potential health risk through dietary fluoride. The risk index of children is higher than that of adults, suggesting their heightened vulnerability to the effects of fluoride pollution. This finding coincides with a study indicating that children face the greatest risks in areas near industrial sites afflicted by fluoride contamination [40]. The reason may be that children’s physical development, particularly that of the liver and kidneys, which play significant roles in metabolism, is not yet fully matured, rendering them more vulnerable. Thus, special attention should be paid to children’s protection against fluoride pollution [24].

For different vegetables, the risk index of fluoride followed the order of leafy vegetables > melon and fruit vegetables > root vegetables in both adults and children (*p* < 0.05), which is consistent with the above results that leafy vegetables have the highest enrichment capacity for fluoride. The risk index of sixteen vegetables in this study area was significantly different (*p* < 0.05). Notably, the risk index of sweet potato leaves reached 1.053 and 1.353 for adults and children, respectively, with health risks. It is recommended that the daily consumption of sweet potato leaves should be controlled in this region.

### 3.4. SSD of Fluoride in Different Species of Vegetables and Ecological Security Threshold of Fluoride Content in the Soil

**Figure 7:**
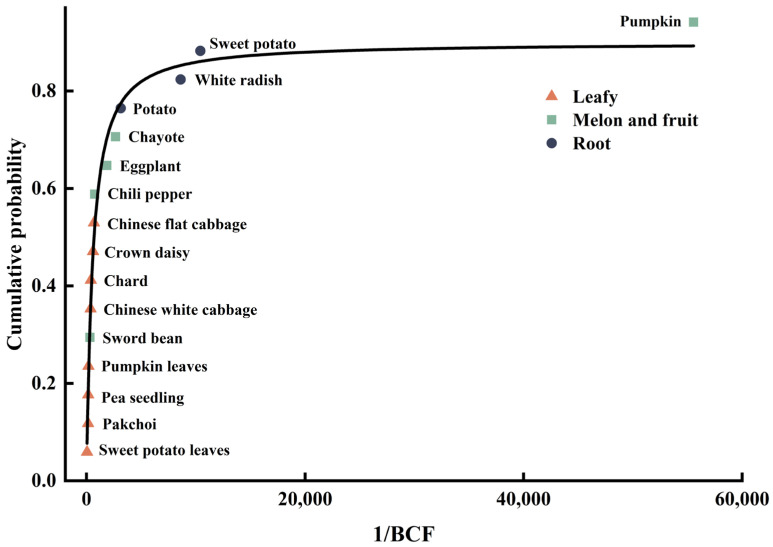
Sensitivity Distribution (SSD) curves of different species of vegetables.

SSD curve showed differences in the sensitivity of different vegetables to fluoride (Figure 7). Pumpkin, sweet potato, white radish, potato, chayote, and eggplant were located at the upper end of the SSD curve, with weak enrichment ability and poor sensitivity to fluoride. Chili pepper, Chinese flat cabbage, crown daisy, chard, Chinese white cabbage, and sword bean were located in the middle of the SSD curve, with the enrichment ability and sensitivity to fluoride at a medium level. Pumpkin leaves, pea seedling, pakchoi, and sweet potato leaves are located at the lower end of the SSD curve and are highly sensitive to fluoride.

Establishing ecological thresholds for soil fluoride can offer theoretical groundwork for categorizing contaminated farmland and implementing efficient strategies to prevent and control soil fluoride pollution. In this study, based on the results of the Log-logistic distribution model and the standard limits for fluoride in vegetables (GB2762-2017), the ecological security thresholds for protecting 20%, 50%, and 80% of vegetables from fluoride contamination of farmland are calculated for local soil scenarios (Table 4). When the fluoride content in the soil ≤ 174.13 mg kg^−1^, 80% of the vegetable species can be protected and the area could be classified as a suitable for planting zone, which is suitable for planting most of the vegetables. When 174.13 mg kg^−1^ < fluoride content in the soil ≤ 696.91 mg kg^−1^, the planting of certain high accumulating vegetables should be restricted, e.g., sweet potato leaves, pakchoi, pea seedling, and pumpkin leaves, while the planting of some medium accumulating vegetables, e.g., chili pepper, Chinese flat cabbage, crown daisy, chard, Chinese white cabbage, and sword bean needs to adopt safe production techniques based on agronomic control. When 696.91 mg kg^−1^ < fluoride content in soil < 4005.42 mg kg^−1^, only some low accumulating vegetable species such as pumpkin, sweet potato, white radish, potato, chayote, and eggplant should be considered for planting, and the synergistic monitoring of fluoride in soil and vegetable products should be strengthened. When the fluoride content in soil ≥ 4005.42 mg kg^−1^, 80% of vegetable species are easily enriched with fluoride. This indicates that the soil can no longer be considered suitable for vegetable cultivation and should be designated as a strictly controlled planting zone. In such areas, alternative crops or ornamental plants should be meticulously chosen based on scientific criteria for cultivation purposes.

For countries that have limited values for fluoride in soil, the criteria vary from hundreds to thousands of mg kg^−1^, owing to variations in derivation method, protection goal and level, data availability, etc. [19]. The Japanese general soil limit value of fluoride is 4000 mg kg^−1^ [41]. The soil limit value of fluoride for resident soil and industrial soil in America is 3100 and 47,000 mg kg^−1^, respectively [31]. The Canada soil quality guideline of fluoride in agricultural soil for the protection of the environment and human health is 200 mg kg^−1^ [42]. In numerous countries, the established limit values for soil quality in agricultural practices rarely consider the diverse soil types and crop cultivars that possess varying capacities for element accumulation. Consequently, it is possible that these limit values may not ensure the safe production of crops [18]. Multiple studies reported contradictory phenomena, such as the inconsistent exceedance of soil samples and the paired crops when using soil standards [21,43]. It is urgent to set an ecological security threshold for soil pollution. The crucial aspect in determining the soil pollution limit value for ecological safety lies in creating an approach that establishes a connection between the bioavailability of elements in soil and the element content found in edible crop organs [20]. Currently, some models have been applied to derive soil ecological security thresholds for heavy metals [44,45,46]. The present study is based on the field dataset and uses the SSD model to derive the threshold values for the farmland soil fluoride; it makes the safe plant utilization of agricultural soil around the PCP in a karst area more specific and accurate. However, field datasets may be subject to various environmental and anthropogenic activities factors that can restrict their applicability. In the future, a more comprehensive study aimed at exploring the scientific validity and rationality of utilizing the SSD method to establish ecological safety thresholds in this specific soil condition should be conducted.

## 4. Conclusions

In this study, fluoride contents in soil and vegetables around a PCP in a karst area were determined to evaluate the contamination status and potential health risks and to derive farmland ecological security threshold. The soil fluoride content of all samples was higher than the Chinese national background value; meanwhile, the median value of fluoride was higher than the mean soil fluoride content in endemic fluorosis areas in China, reaching pollution levels. The fluoride content in 53.85% of vegetable samples exceeded the limits of contaminants in food, and the four leafy vegetables (sweet potato leaves, pakchoi, pumpkin leaves, and pea seedling) had the strongest enrichment ability to fluoride. Despite that, there is no significant health risk to the residents via the intake of vegetables overall. However, it is worth noting that there may be potential health risks from the intake of sweet potato leaves. Based on the SSD model, the ecological safety thresholds of fluoride in local farmland were classified as suitable for planting category (≤174.13 mg kg^−1^), safe plant utilization category (174.13–4005.42 mg kg^−1^), and strict control of planting category (≥4005.42 mg kg^−1^). This study provides an important scientific basis for the prevention and control of soil fluoride pollution and the safe cultivation of vegetables.

## Figures and Tables

**Table 1:** Different vegetables in the study area.

Vegetable Specie	Vegetable Type	Number
Pakchoi (*Brassica chinensis* L.)	Leafy	8
Pea seedling (*Pisum sativum* L.)	Leafy	3
Crown daisy (*Chrysanthemum coronarium* L.)	Leafy	3
Chard (*Beta vulgaris var. cicla* L.)	Leafy	3
Pumpkin leaves (*Cucurbita moschata* Duch.)	Leafy	3
Sweet potato leaves (*Ipomoea batatas* L.)	Leafy	6
Chinese white cabbage (*Brassica napus* L.)	Leafy	19
Chinese flat cabbage (*Brassica narinosa* L.)	Leafy	3
Eggplant (*Solanum melongena* L.)	Melon and fruit	5
Pumpkin (*Cucurbita moschata* Duch.)	Melon and fruit	3
Chili pepper (*Capsicum annuum* L.)	Melon and fruit	10
Chayote (*Sechium edule* (*Jacq.*) Swartz)	Melon and fruit	3
Sword bean (*Canavalia gladiata* (*Jacq.*) DC.)	Melon and fruit	3
Potato (*Solanum tuberosum* L.)	Root	3
White radish (*Raphanus sativus* L.)	Root	13
Sweet potato (*Ipomoea batatas* L.)	Root	3

**Table 2:** Evaluation standards of soil fluoride environment quality.

Classification	Fluoride Content	P_i_	Pollution Level
Ⅰ	Fluoride content ≤ 478 mg kg^−1^	P_i_ ≤ 0.6	Clean
Ⅱ	478 mg kg^−1^ < Fluoride content ≤ 800 mg kg^−1^	0.6 < P_i_ ≤ 1	Warning
Ⅲ	Fluoride content > 800 mg kg^−1^	1 ≤ P_i_	Pollution

**Table 3:** pH and fluoride contents in the soil at different sites.

Sampling Site	Distance from Source (km)	pH					Fluoride (mg kg^−1^)				
		Mean	SD	Median	Min.	Max.	Mean	SD	Median	Min.	Max.
A (n = 8)	0.5	4.67	0.39	4.54	4.34	5.48	935.45	151.29	863.68^a^	824.35	1249.61
B (n = 8)	1	5.62	0.84	5.41	4.80	6.84	873.69	298.22	823.93^a^	561.71	1496.02
C (n = 8)	2	5.83	1.28	5.56	3.97	7.40	859.29	216.76	805.19^a^	649.73	1249.61
D (n = 8)	3	5.94	0.88	6.00	4.86	7.22	609.38	81.58	580.93^b^	529.36	749.77
Total		5.52	1.00	5.21	3.97	7.40	819.45	231.07	823.93	529.36	1496.02

Different letters indicate significant differences between sampling sites (*p* < 0.05).

**Table 4:** Classification threshold of soil fluoride environmental quality in vegetables-producing areas.

Q/%	Soil Ecological Division Threshold/mg kg^−1^	Soil Quality Category for Planting
80	174.13	Suitable for planting
50	696.91	Safe plant utilization
20	4005.42	Strict control of planting

Q of 20%, 50%, and 80% represent the ecological security thresholds of soil fluoride that protects 20%, 50%, and 80% of vegetable species, respectively.

## Data Availability

The data presented in this study are available on request from the corresponding author.
